# Bacteria associated with periodontal disease are also increased in health

**DOI:** 10.4317/medoral.23766

**Published:** 2020-07-23

**Authors:** Jesús López-Martínez, Natalia Chueca, Miguel Padial-Molina, Jose Angel Fernandez-Caballero, Federico García, Francisco O’Valle, Pablo Galindo-Moreno

**Affiliations:** 1 Department of Oral Surgery and Implant Dentistry, School of Dentistry, University of Granada, Spain; 2 Microbiology and Parasitology Laboratory, University Hospital, Granada; 3 Department of Pathology, School of Medicine, University of Granada; 4 Institute of Biopathology and Regenerative Medicine (IBIMER, CIBM), University of Granada; 5 Instituto Biosanitario de Granada (ibs.GRANADA), University of Granada

## Abstract

**Background:**

The objective of this cross-sectional clinical study was to analyze the differences in the microbiome in gingival sulci of adult patients in the presence or absence of chronic periodontitis.

**Material and Methods:**

Patients with or without periodontal disease were included in this cross-sectional study. Subgingival biofilm samples were collected and analyzed by 16S massive pyrosequencing. Functional analyses were also performed.

**Results:**

A total of 15 phyla, 154 genera and 351 species were detected globally. Differences between disease and non-disease samples were observed in all taxonomical levels which suggest functional profile changes in the community. It was found that the main species associated with non-disease samples were reduced in disease but not completely suppressed. Analysis of the functional potential of the biofilms revealed a significantly higher activity related to endocytosis and phosphatidylinositol signaling in the disease group but lower cell adhesion molecules.

**Conclusions:**

Specific differences between health and disease suggest functional profile changes in the community, although bacteria associated with periodontal disease are also increased in health. Transcriptome studies should be conducted to confirm and deepen metabolic dysfunctions.

** Key words:**Pyrosequencing, 16S rRNA, oral microbiome, periodontitis, functional potential.

## Introduction

Periodontitis is an inflammatory disease induced by biofilm and characterized by bone loss around the dentition (1). The microbial plaque is required to induce the inflammatory response but it is not sufficient to induce periodontitis (2). Thus, a complex balance is responsible to maintain health.

The oral microbiota is composed by a combination of viruses, protozoa, fungi, archaea and bacteria. Historically, the composition of subgingival microflora has been characterized by culture methods. However, culture techniques present important drawbacks. For instance, only viable bacteria can grow in specific culture media and strict sampling and transporting conditions are essential. Furthermore, if non-selective media is used, the sensitivity of culturing bacteria can be particularly low, with limits of detection averaging 103–104 bacterial cells. Thus, high numbers of a specific bacteria in a sample are needed to allow its detection (3).

Using molecular investigations like whole-genomic hybridization, Socransky *et al*. stablished a strong association between *Porphyromonas *gingivalis**, *Treponema denticola* and *Tannerella forsythia* and periodontal disease and with each other (4). This set of three is among over 1000 bacterial species that may reside in the mouth. Of those, individuals may carry over 200 species in their oral microbiome at a given time (5). Additionally, the progression in identifying associations between microbes and dental diseases with the use of species-specific 16S primers for PCR amplification have provided a large amount of new data. The 16S is the most used macromolecule in microbial phylogeny and taxonomy. By sequencing it, the bacteria can be unequivocally identified; by sequencing all 16S genes in a population, it is possible to describe the ecological site and the number of each one of the components. Initial reports exploiting cloning and Sanger sequencing did not provide sufficient information to unravel the complexity of the microbial ecosystem at an economical approachable level (6). The advent of the 454 pyrosequencing of 16S rRNA genes helped to overcome this limitation as it allows the compilation of thousands of sequences per sample. With this method, bacterial community composition can be studied at the level of species. Thus, it should always be used in new studies of the periodontal and peri-implant microbiology (7,8). Even more, besides species abundance, it is currently possible to predict a snap-shot of the functional hypothetical capacities of a microbial community by analyzing its 16S taxonomic abundance. Vikodak is one of the available platforms available to do so (9).

Therefore, the objective of this clinical study was to analyze the differences in the microbiome in teeth of adult patients in the presence and absence of chronic periodontitis by using techniques of pyrosequencing the 16S rRNA gene and to compare their functional profiles.

## Material and Methods

- Study design and patients

This clinical cross-sectional study included a total of 10 sites with periodontally healthy dentition and 12 sites with chronic periodontitis attending the School of Dentistry, University of Granada (Approval number 2/CEIH/2015 from the Ethics Committee for Human Research of the University of Granada). Informed consent was obtained from all individual participants included in the study.

The healthy group had a minimum of 20 teeth with no bleeding on probing (BoP) and no more than 4 mm of clinical attachment loss (CAL). To be included in the periodontitis group, subjects had to have at least 4 mm of CAL and more than 5 mm probing depth (PD) in at least three non-adjacent interproximal sites and more than 20% of sites with BoP. None of the patients were smokers nor affected by systemic diseases, under antibiotic therapy or subjected to dental cleaning in the last 6 months. Plaque Index (PI), Gingival Index (GI), PD, gingival margin (GM) and BoP at six sites per tooth (mesiobuccal, buccal, distobuccal, mesiolingual, lingual, and distolingual) were recorded.

- Sample collection

Plaque samples were obtained from the subgingival space of the periodontal pocket. The procedure was initiated by isolating the area with cotton rolls. Then, saliva and excess of fluid were removed by gentle air. A sterile paper point was then inserted into the sulcus/pocket for 30 secs when it was immediately transferred to an Eppendorf tube and stored at -80ºC until further processing.

- DNA extraction and pyrosequencing

The microbes were extracted and their DNA amplified and cloned by emPCR. Briefly, the DNA was extracted by using the QIAmp® DNA Mini Kit (Qiagen GMBH, Hilden, Germany). Then, a 600-bp sequence in the V1-V3 region of the 16S rRNA gene was amplified by using barcoded primers in a total volume of 15 µl for each sample. As recommended by the sequencer manufacturer, a mix was prepared containing the primers 27F (10 µmol/l), 534R (10µmol/L), dNTP mix (10 mmol/L), FastStart 10× buffer (18 mmol/L of MgCl2), FastStart HiFi polymerase (5 U in 1 mL), and 2 µL of genomic DNA. The mix was included in a FastStart High Fidelity PCR System (Roche Applied Science, Penzberg, Germany) set to perform one cycle of 95°C for 2 minutes, 30 cycles of 95°C for 20 seconds, 56°C for 30 seconds, and 72°C for 5 minutes, and final step at 4°C. After PCR, smaller fragments were removed by using AMPure XP beads (Beckman-Coulter, CA, USA). Finally, DNA quality and concentration were measured using a Quant-iT™ PicoGreen® dsDNA Assay Kit (Invitrogen™, Thermo Fisher Scientific Inc., MA, USA) and the PCR amplicons pooled in equimolar ratios to create a DNA pool (109 DNA molecules) that was utilized for clonal amplification (emPCR) and pyrosequencing by using a Roche/454 GS Titanium technology platform (Branford, CT, USA). Then, a quality check was conducted to remove any poor quality and short reads.

- Taxonomic analysis

First, sequences <150 bp, any non-16S bacterial reads, mitochondrial DNA, plasmids, chimeras, primers and barcodes were trimmed. MG-RAST (http://www.mg-rast.org/) with the Ribosomal Database Project (RDP) were used to analyze all sequences following demultiplexing, quality filtering, length filtering, dereplication, and removal of model organism sequences. Then, FASTQ sequences were filtered using a dynamic trimming. The specific lowest Phred quality score counted as a high-quality base was 15 so that all sequences containing 5 bases below that value were trimmed.

- Functional analysis

The functional potential profiles were obtained and compared against the Vikodak database (http://metagenomics.atc.tcs.com/vikodak). Briefly, as designed, the Vikodak platform uses the 16S metagenomic dataset to estimate the relative abundance of different metabolic pathways, quantify how each microbe contributes to the previous estimation and identify the core of functions of the whole environment (9). With such information, a pair-wise comparison was then performed.

- Statistical analysis

For the metagenomic analysis, the STAMP software (version 2.1.3.) was used. SPSS Statistics (version 20.0; SPSS Inc.) was used to analyzed the MG-RAST output file. Differences between groups were evaluated with the Mann-Whitney U test and chi-square test. Graphics were designed with SPSS and Graphpad Prism (version 7). In all cases, significance was accepted at *p*<0.05.

## Results

Demographic data is presented in Table 1.

**Table 1:**
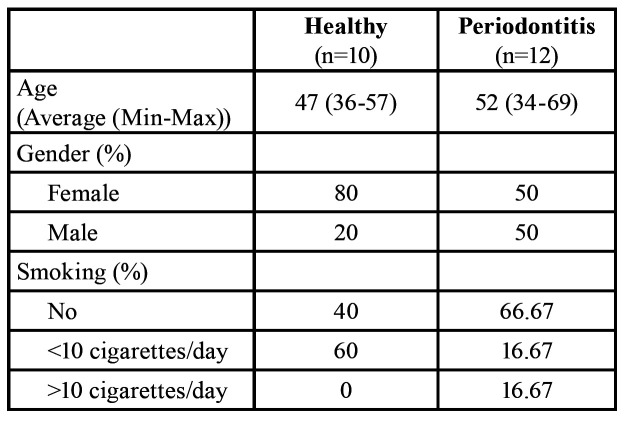
Demographic data.

**Figure 1:**
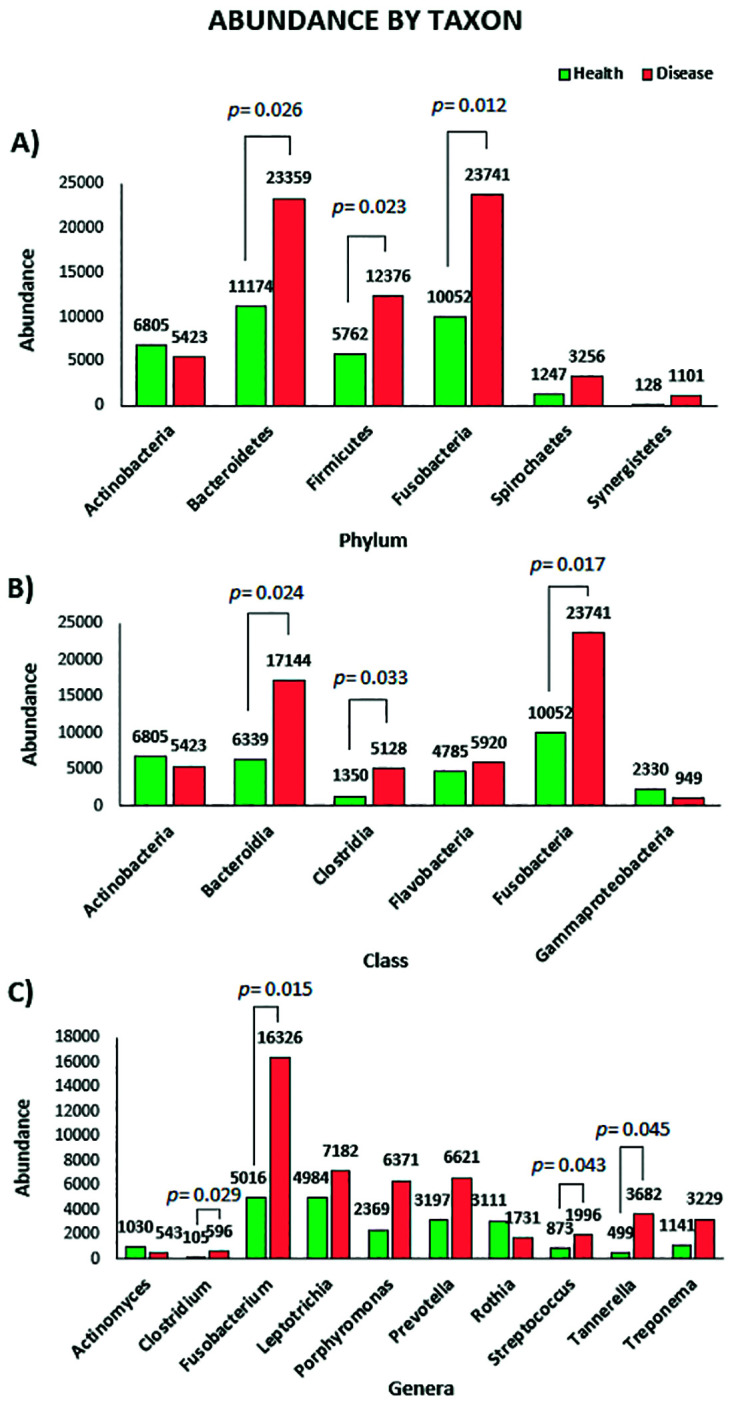
Bar graphs representing the microbial abundance by A) Phylum, B) Class and C) Genus both in health (green) and disease (red).

**Figure 2:**
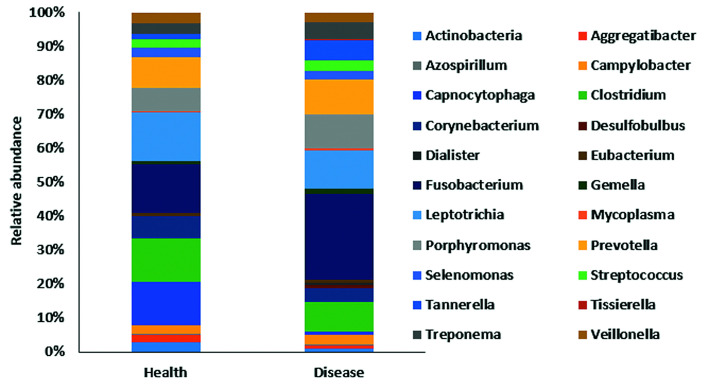
Graph of distribution by genera identifying differences in health and disease.

With a total of 80000 reads from periodontal disease samples and 44000 from health, a total of 15 phyla, 154 genera and 351 species were detected globally. All abundance values were normalized. Overall, differences between samples from healthy and periodontally diseased teeth were observed in all taxonomical levels. By phylum (Fig. 1), *Bacteroidetes*, *Firmicutes*, *Fusobacteria*, Spirochaetes and *Synergist*ete were highly associated with periodontal disease. On the other hand, *Actinobacteria* was found in higher proportion in health. By class (Fig. 1), Bacteroidia, Clostridia, Flavobacteriia and Fusobacteriia were found in higher proportion in periodontal disease, while Gammaproteobacteria was more abundant in health. By genera (Fig. 1), *Fusobacterium*, *Leptotrichia*, *Porphyromonas*, *Prevotella*, *Tannerella* and Treponema were more frequently detected in disease and *Actinomyces* and *Rothia* were more frequently detected in health. A phylogenetic tree of relative abundance rebuilt as two bars by genera was generated (Fig. 2; colors represent class).

The ecological analysis showed high average alpha diversity (within each site) in periodontal disease (26.36, min-max: 11.38-56.77) than in health (22.96, min-max: 8.38-35.13), although not statistically significant (*p*=0.497). Measuring this diversity by the Shannon (5.38 vs. 4.16, health vs. disease; *p*=0.032) and inverse Simpson (6.52 vs 2.23 health vs. disease; *p*=0.014) indexes, it was confirmed that it was higher in the health group with significant values. In addition, the beta diversity (between communities) was also calculated using the formula B_w=(b+c)/(2a+b+c), where “a” is the number of species shared between sites, and “b” and “c” are the number of species that are unique per site. This index was calculated to be 0.32 (unique species in the periodontitis group=57; unique species in the healthy group=112; shared species=239). Thus, comparison between sites reflects higher variability in health while periodontitis sites were more similar between themselves.

**Figure 3:**
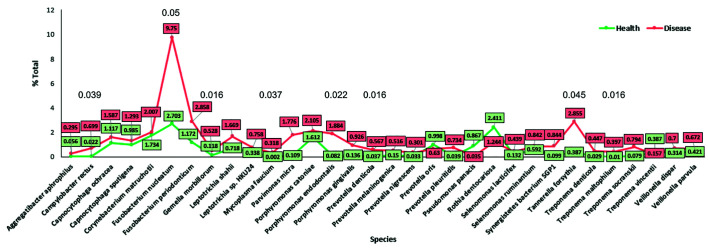
Differences between health and disease at the species levels. Data on abundance are presented as percentage. Only those genera or species with ≥0.1% or ≥0.2% difference, respectively, between health and disease are shown in the graph.

Relative abundance in percentage of species between healthy and disease samples for separate SubG are shown in Fig. 3, considering the most abundant species with significant differences between health and disease (*p*<0.05). At the species level, more *Fusobacterium nucleatum* and *Tannerella forsythia* were found in disease. In health, *Rothia* dentocariosa was found more abundantly. It was found that the main species associated with health were reduced in disease but not completely suppressed. Also, bacteria associated with periodontal disease were also increased in health. The statistical analysis showed a total of 3 phyla, 4 genera and 10 species that were differently abundant in health and disease (*p*<0.05).

**Figure 4:**
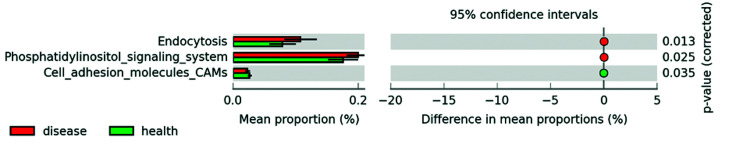
Summary figure demonstrating statistically significant differences in functionality of species associated to health and disease and corrected <italic>p</italic> values.

Analysis of the functional profiles showed statistically significant higher activity related to endocytosis (0.129±0.031 vs. 0.095±0.025; *p*=0.013, CI 95% [0.008, 0.060]) and phosphatidylinositol signaling (0.239±0.022 vs. 0.210±0.029); *p*=0.024, CI 95% [0.004, 0.054) in the disease group but lower cell adhesion molecules (0.028±0.005 vs. 0032±0.004); *p*=0.035, CI 95% [-0.008, -0.0003]) (disease vs. healthy groups) (Fig. 4).

## Discussion

In the last decade, a tremendous effort is ongoing to generate a complete mapping of the human microbiome under the Human Microbiome Project (HMP) initiative. So far, it has been demonstrated that, of all bacteria in the human body, approximately 26% are located in the oral cavity (10). Thus, the oral cavity is a complex microbial environment not only by itself but, more importantly, in the interaction with the human physiology (11). The most commonly found diseases in the oral cavity and maybe even in humans as a species are those that involve the tissues surrounding the teeth (12). Therefore, understanding the pathological development of these diseases requires an understanding of the microbiology that triggers the host response that will ultimately be reflected clinically.

The synergistic activities between the periodontal tissues and microbes must be evaluated from an ecological point of view, including the stability and changes of the microbiota within individuals and over time as well as between families and communities both in health and disease (13). These studies can only be carried out by using deep analysis tools like high-density microarrays, targeted large-insert clone sequencing and random shotgun sequencing procedures.

Within the specific fields of Dentistry and Periodontics, the use of pyrosequencing is providing new insights into the pathogenesis of the periodontal disease and other oral diseases such as caries and peri-implant diseases. For example, the periodontal microbiota has been found to be more diverse than previously thought (14). As previously reviewed (15) and confirmed in the current study, proportions of some genera and species are clearly distinct between health and disease. However, other genera and species have been identified as supporters of the community and referred to as keystones (16) that would ultimately contribute to the ecological catastrophe defined by Marsh (17). Some of the keystones reported in the literature could not be confirmed in the present study. Our results indicate that *P. *gingivalis** (in disease) and R. dentocariosa were elevated only in very few patients. However, there might be other less common microbes that would in fact be the key for changing the environment, pre-processing nutrients, enhance protection from host defenses and even triggering initial host-responses that would ultimately allow the development of the disease (18). Moreover, some of these microbes are “inflammophilic”: they have evolved to deal with inflammation and to take advantage of it (2). These fewer common bacteria include *Peptostreptococcus* and Filifactor (higher in disease) and *Streptococcus* and *Veillonella* (higher in health). In this sense, some key concepts have been summarized into the “Polymicrobial synergy and dysbiosis” hypothesis of periodontal disease pathogenesis (19). Briefly, this hypothesis proposes that periodontitis is initiated by a synergistic and dysbiotic microbiota that shape and stabilize a disease-provoking microbiota by disrupting the inflammatory response to take advantage of the new local environment that inhibits certain type of competing bacteria. This, in turns, provides tissue breakdown products as key nutrients. In the current study, we have also found that these changes in the community potentially reflect functional changes in the environment. Furthermore, in the current study, the main species associated with health were reduced in disease but not completely suppressed, which correlates with previous similar studies (20).

Overall, higher abundance and diversity are found in periodontal disease (20). However, the contrary has likewise been reported (21), as we have also found. Although still to be confirmed, these differences could be due to the different populations under study, either Asian (21) or Caucasian (20). In addition, particular clinical parameters are yet to be correlated with the potential of the pyrosequencing technology. Age and sex have not been correlated with the microbial community patterns (21). However, smoking, for example, is associated with a higher diversity and disease-associated community in comparison to never-smokers, even in healthy patients (22). In the current study, the specific correlation between microbiological data and clinical parameters was not analyzed.

The current study has identified specific differences on important aspects that are arising in the literature, such as the functional profile of the biofilm. Studies on these aspects are still very limited in the periodontal field (23). These new approaches for understanding what microorganisms are doing rather than who they are have been recommended to improve the understanding of the functions of the oral microbiome (24). Using this “metatranscriptome” approach, new analyses are highlighting the complexity of these interactions. So far, periodontal pathogens have been associated *in vitro* with an upregulation of transcription factors related to growth and division (25). In clinical studies, periodontal disease has been associated to increased activities of pyruvate and lysine fermentation, histidine catabolism, and nucleotide biosynthesis (26), as well as synthesis of cytotoxic short-chain fatty acid butyrate (27). It has also been found that, during disease, rather than oxidative pathways to energy use, the biofilm shifts to fermentation and methanogenesis processes (28) and iron acquisition, lipopolysaccharide synthesis and flagellar synthesis (29). In fact, although differences in composition can be found in healthy between patients, communities associated with disease environments have been reported to display similar metabolic profiles despite being taxonomically different (26). Thus, rather than particular microbes, the whole community is responsible for inducing specific metabolic activities (11). With the current study, we add supporting information to these previous observations. We have found significantly increased endocytosis and phosphatidylinositol signaling in diseased patients but less cell adhesion molecules. However, because the available literature on these aspects is still limited, we have not been able to clarify how the modification of the mentioned functional profiles contributes to the disease state. This information has not been generated by studying miRNA or the transcriptome of the samples. In turn, it has been inferred by analyzing the whole 16S metagenome. Although the presence of the gene in the metagenomic pool does not mean that they are expressed, the analysis of the potentiality of the microbes in an environment is still important (30).

One of the main problems of the reported studies is the sample size, ranging from 12 to 16. In the current study, we increased the sample size to a total of 22, which, as far as we know, is among the largest on this topic until today.

## Conclusions

The current study has found different microbial diversity and abundance in health and periodontal disease patients and a significantly different pattern on tooth microbiota distribution. Interestingly, the species associated with health are not completely depleted in disease but there is a change in their proportions. In addition, their functional profiles are also different, although additional studies should be conducted to deepen the importance of some specific functional potentials. Particular differences associated with specific periodontal clinical characteristics are yet to be analyzed.
